# *Amauroderma rugosum* Extract Suppresses Inflammatory Responses in Tumor Necrosis Factor Alpha/Interferon Gamma-Induced HaCaT Keratinocytes

**DOI:** 10.3390/molecules27196533

**Published:** 2022-10-03

**Authors:** Polly Ho-Ting Shiu, Jingjing Li, Chengwen Zheng, Panthakarn Rangsinth, Renkai Li, Queenie Tze-Lam Cheung, Angel Heng-Yee Lau, Jacqueline Cho-Ki Chan, Yiu-Wa Kwan, Timothy Man-Yau Cheung, George Pak-Heng Leung

**Affiliations:** 1 Department of Pharmacology and Pharmacy, The University of Hong Kong, Hong Kong 999077, China; 2 Department of Rehabilitation Sciences, Faculty of Health and Social Sciences, Hong Kong Polytechnic University, Hong Kong 999077, China; 3 School of Biomedical Sciences, Faculty of Medicine, The Chinese University of Hong Kong, Hong Kong 999077, China; 4 Tian Ran Healthcare Limited, Hong Kong 999077, China

**Keywords:** *Amauroderma rugosum*, anti-inflammatory, keratinocytes, gallic acid, nucleosides

## Abstract

Keratinocytes form the physical barrier of the skin and play an important role in the inflammatory process. *Amauroderma rugosum* is an edible mushroom; however, its pharmacological properties have seldom been studied. Although the anti-inflammatory effect of the organic solvent extract of *Amauroderma rugosum* has been previously reported, it is not known whether the aqueous extract has a similar effect. In addition, the effect of *Amauorderma rugosum* extract on skin has never been explored. Therefore, the objectives of the present study were to evaluate the anti-inflammatory effects of the aqueous extract of *Amauroderma rugosum* on HaCaT keratinocytes, to explore its mechanisms of action, and to study the possible active ingredients involved. The results showed that the aqueous extract of *Amauroderm rugosum* at a concentration of 1.5 mg/mL was non-toxic to HaCaT cells and inhibited the release of cytokine interleukin-1β, and chemokines interleukin-8 and monocyte chemoattractant protein-1 in tumor necrosis factor (TNF)-α- and interferon (IFN)-γ-stimulated HaCaT cells. *Amauroderma rugosum* extract reduced the intracellular levels of reactive oxygen species. In addition, *Amauroderma rugosum* extract reduced the total protein expression of nuclear factor-kappa B (NF-κB) and B-cells inhibitor alpha in HaCaT keratinocytes and inhibited the phosphorylation of mitogen-activated protein kinase kinase (MEK) 1/2, extracellular signal-regulated kinase (ERK) 1/2, protein kinase B (Akt), and mammalian target of rapamycin (mTOR) in TNF-α- and INF-γ-stimulated HaCaT keratinocytes. Chemical analysis revealed that the aqueous extract of *Amauroderma rugosum* contains polysaccharides, triterpenes, and phenolic compounds. Anti-inflammatory compounds, such as gallic acid, guanosine, and uridine, were also present. The anti-inflammatory effect of *Amauroderma rugosum* could be mimicked by a combination of gallic acid, guanosine, and uridine. In conclusion, our study suggests that the aqueous extract of *Amauroderma rugosum* exerts anti-inflammatory effects on keratinocytes through its antioxidant and inhibitory effects on MEK/ERK-, Akt/mTOR-, and NF-κB-dependent signaling pathways.

## 1. Introduction

It is well-established that keratinocytes, which comprise 95% of the human epidermis, play an important role in the immune response of the skin. Upon stimulation by allergens and microbiological agents, keratinocytes express and release pro-inflammatory cytokines, chemokines, and adhesion molecules, which leads to skin inflammation through increased immunocyte infiltration at inflammatory sites [1]. Moreover, several skin diseases are initiated and exacerbated by oxidative stress. Increased oxidative stress triggers the release of pro-inflammatory cytokines, which leads to chronic low-grade inflammation and causes the further production of reactive oxygen species (ROS) [2], thus forming a vicious cycle.

*Amauroderma rugosum* (AR) is a basidiomycete belonging to the *Ganodermataceae* family. Although AR has not been widely studied, existing literature has reported its antioxidant and anti-inflammatory effects. Different extracts of AR have been shown to exhibit DPPH and ABTS scavenging activities in in vitro models [3,4,5,6,7]. In addition, AR inhibits nitric oxide production in lipopolysaccharide-stimulated RAW264.7 and BC-2 microglial cells [3,4,8]. In lipopolysaccharide-stimulated RAW264.7 cells, AR downregulates the expression of pro-inflammatory cytokines such as tumor necrosis factor (TNF)-α, while upregulating the expression of anti-inflammatory cytokines such as IL-10 [4]. AR contains sterols, flavonoids, fatty acids, aromatic acids, phenolic compounds, polysaccharides, and triterpenes [9]. Interestingly, the antioxidant effect of AR is higher than that of *Ganoderma lucidum*, which is the most common and widely studied mushroom in the *Ganodermataceae* family. One possible reason for this is the higher content of phenolic compounds in AR [10].

In addition to its antioxidant and anti-inflammatory effects, AR also possesses anti-cancer, anti-hyperlipidemic, antibacterial, and neuroprotective effects [9]. Organic solvents such as methanol and ethyl acetate have been used for the extraction of AR in most of the aforementioned studies. However, pharmacological studies on aqueous extracts, which are economical, efficient, and simple methods for the extraction of herbal medicines, are rarely reported. In addition, the effects of AR on keratinocytes have not been explored. Therefore, the present study aimed to evaluate the anti-inflammatory effects of an aqueous extract of AR on tumor necrosis factor (TNF)-α/interferon (IFN)-γ-stimulated HaCaT keratinocytes. The mechanisms of action and active ingredients of AR were also investigated.

## 2. Results

### 2.1. Chemical Analysis of Aqueous Extract of AR

Total polysaccharides, triterpenes, and phenolic compounds in the AR extract were measured using chemical assays. The content of total polysaccharides in AR extract was 44.23 ± 3.17  mg GE/g of dry weight. The content of total triterpenes was 5.89 ± 0.13  mg OAE/g of dry weight. The content of total phenolic compounds of AR extract was 6.82 ± 0.21 mg GAE/g of dry weight.

The chemical constituents of the aqueous extract of AR were identified using mass spectrometry (Table 1). The quantities of gallic acid, guanosine, and uridine in the aqueous extract of AR were measured by UPLC. The results showed that the content of gallic acid, guanosine, and uridine were 1.22 ± 0.13 mg/g, 0.95 ± 0.14 mg/g, and 1.13 ± 0.15 mg/g dry weight, respectively.

### 2.2. Effects of AR Extract on Viability of HaCaT Keratinocytes

**Figure 1:**
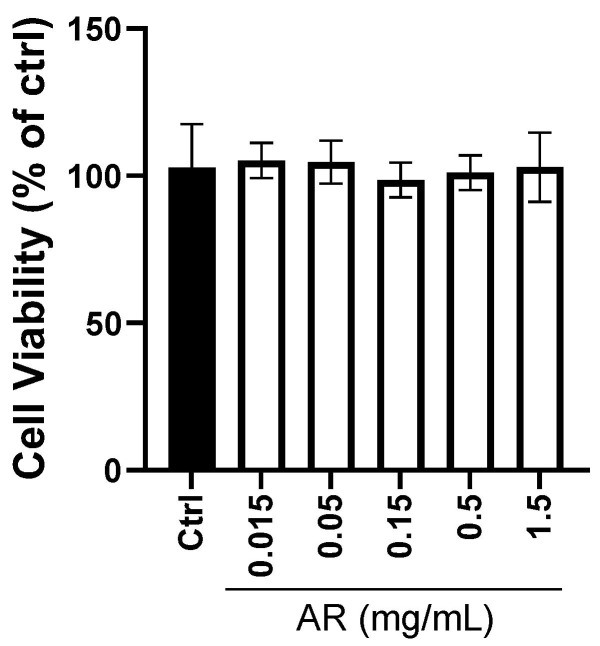
Effect of *Amauroderma rugosum* (AR) extract on the viability of HaCaT keratinocytes. HaCaT cells were treated with various concentrations of AR extract (0.015 to 1.5 mg/mL) or vehicle (control) for 24 h. Cell viability was determined using the MTT assay. Data are presented as the percentage of control group values (mean ± SD of three independent experiments).

MTT assays showed that AR extract at concentrations lower than 1.5 mg/mL did not affect the viability of HaCaT keratinocytes (Figure 1). Therefore, this concentration range from 0.015 to 1.5 mg/mL of AR extract was used in subsequent experiments.

### 2.3. Effects of AR Extract and Its Possible Active Ingredients on Pro-Inflammatory Cytokine and Chemokine Release from HaCaT Keratinocytes

**Figure 2:**
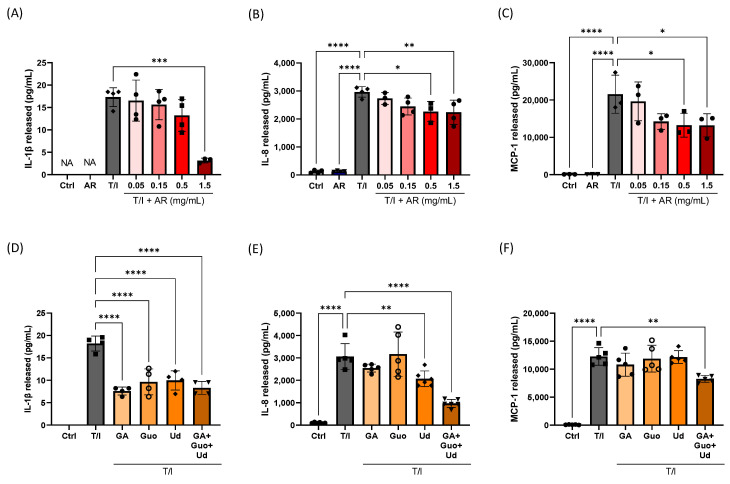
Effects of *Amauroderma rugosum* (AR) extract on cytokine and chemokine release from HaCaT keratinocytes. HaCaT cells were treated without (control) and with different concentrations of (**A**–**C**) AR extract (0.05 to 1.5 mg/mL), or (**D**–**F**) gallic acid (GA; 10 µM); guanosine (Guo; 5 µM), uridine (Ud; 5 µM); or a combination of gallic acid (10 µM), guanosine (5 µM), and uridine (5 µM), in the presence of 10 ng/mL recombinant human TNF-α and 10 ng/mL IFN-γ (T/I) for 24 h. IL-1β, IL-8, and MCP-1 in the culture media were detected using ELISA. Data are presented as a percentage of control group values (mean ± SD of three to six independent experiments). **** *p* < 0.0001, *** *p* < 0.001, ** *p* < 0.01, and * *p* < 0.05 indicate a significant difference.

The effects of AR extract on the pro-inflammatory cytokine IL-1β, and chemokines IL-8 and chemoattractant protein-1 (MCP-1) were evaluated. TNF-α/IFN-γ could increase IL-1β, IL-8, and MCP-1 release in HaCaT keratinocytes by 240%, 2372%, and 21,302%, respectively. AR extract itself had no effect on IL-8, IL-1β and MCP-1. However, 1.5 mg/mL AR extract significantly reduced TNF–α/IFN-γ-induced IL-1β release by 82%. AR extract (0.5 and 1.5 mg/mL) reduced TNF-α/IFN-γ-induced IL-8 release by 24% and 25%, respectively. AR extract (0.5 and 1.5 mg/mL) inhibited TNF-α/IFN-γ-induced MCP-1 release by 38% and 39%, respectively (Figure 2A–C).

Further experiments were performed to determine the possible ingredients responsible for the anti-inflammatory effect of the AR extract. According to the results of chemical analysis, 1.5 g/mL of AR extract contained 10.7 ± 1.1 µM gallic acid, 6.1 ± 0.9 µM guanosine, and 6.9 ± 1.3 µM uridine. These concentrations of gallic acid, guanosine, and uridine showed a decrease in IL-1β release by 36%, 25%, and 23%, respectively, but failed to affect the release of MCP-1 (Figure 2D–F). Uridine could inhibit IL-8 release by 32%, whereas gallic acid and guanosine had no effect on IL-8 release. When the cells were incubated with a combination of gallic acid, guanosine, and uridine, the TNF-α/IFN-γ-induced IL-1β, IL-8, and MCP-1 release were decreased by 54%, 68%, and 33%, respectively (Figure 2D–F).

### 2.4. Antioxidant Effects of AR Extract on HaCaT Keratinocytes

**Figure 3:**
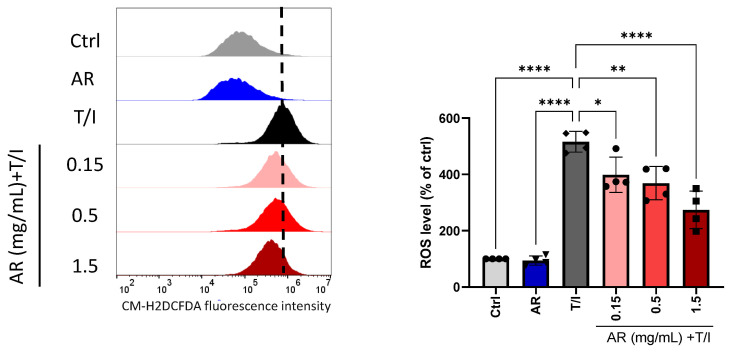
Effect of *Amauroderma rugosum* (AR) extract on ROS generation in HaCaT keratinocytes. HaCaT cells were treated without (control) and with different concentrations of AR extract (0.15 to 1.5 mM)) in the presence of 10 ng/mL recombinant human TNF-α and 10 ng/mL IFN-γ (T/I) for 24 h. ROS generation in HaCaT cells was detected by CM-H2DCFDA staining and quantified via flow cytometry. Data are presented as a percentage of control group values (mean ± SD of four independent experiments). **** *p* < 0.0001, ** *p* < 0.01, and * *p* < 0.05 indicate a significant difference.

AR extract itself had no effect on ROS generation, but the ROS level was elevated significantly by 429% after treatment with TNF-α/IFN-γ. The TNF-α/IFN-γ-induced ROS was reduced by 0.15, 0.5, and 1.5 mg/mL AR extract by 22%, 28%, and 46%, respectively (Figure 3).

### 2.5. Effects of AR Extract on the Inflammation-Related Signalling Pathways in HaCaT Keratinocytes

**Figure 4:**
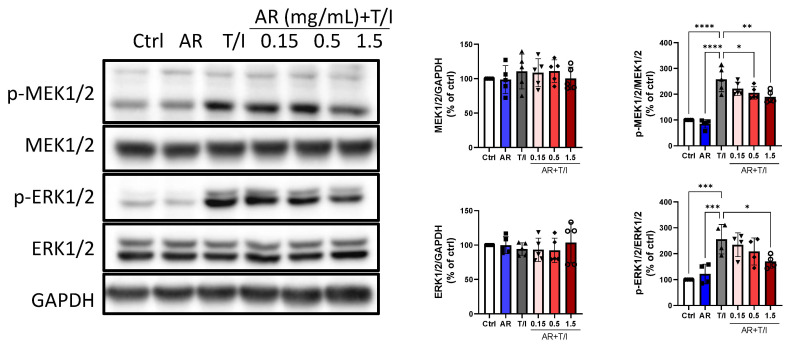
Effects of *Amauroderma rugosum* (AR) extract on total and phosphorylated protein levels of MEK1/2 and ERK1/2 in HaCaT keratinocytes. HaCaT cells were treated without (control) and with different concentrations of AR extract (0.15 to 1.5 mg/mL) in the presence of 10 ng/mL recombinant human TNF-α and 10 ng/mL IFN-γ (T/I) for 24 h. Protein expression levels of MEK1/2, p-MEK1/2, ERK1/2, and p-ERK1/2 in HaCaT cells were determined using Western blotting analysis. GAPDH was used as a reference and the amounts of different proteins were normalized to that of GAPDH. Data are presented as a percentage of control group values (mean ± SD of three to six independent experiments). **** *p* < 0.0001, *** *p* < 0.001, ** *p* < 0.01, and * *p* < 0.05 indicate a significant difference.

Several signal transduction pathways are involved in the inflammatory response of keratinocytes. Western blot analysis was performed to study the effects of the AR extract on inflammation-related signaling pathways. TNF-α/IFN-γ did not affect the expression levels of total mitogen-activated protein kinase (MAPK) kinase (MEK)1/2 and extracellular signal-regulated kinase (ERK)1/2 but could increase their phosphorylation levels by 158% and 156%, respectively. AR extract (1.5 mg/mL) did not affect the total and phosphorylated protein expression of MEK1/2 and ERK1/2, but 0.5 and 1.5 mg/mL AR extract reduced the TNF-α/IFN-γ-induced phosphorylation levels of MEK1/2 by 20% and 26%, respectively. Moreover, 1.5 mg/mL AR extract reduced the TNF-α/IFN-γ-induced phosphorylation of ERK1/2 by 34% (Figure 4).

**Figure 5:**
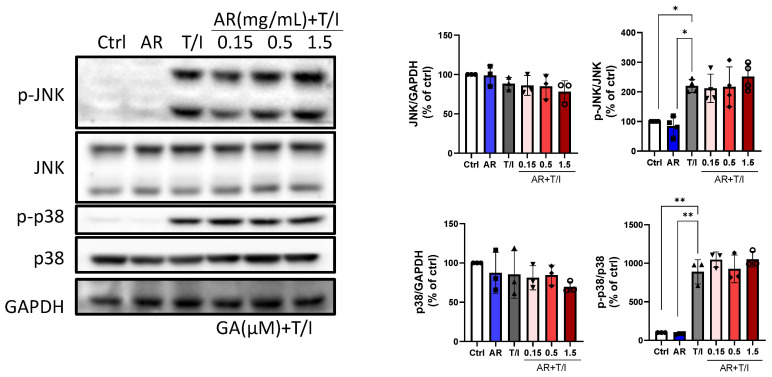
Effects of *Amauroderma rugosum* (AR) extract on total and phosphorylated protein levels of JNK and p38 MAPK in HaCaT keratinocytes. HaCaT cells were treated without (control) and with different concentrations of AR extract (0.15 to 1.5 mg/mL) in the presence of 10 ng/mL recombinant human TNF-α and 10 ng/mL IFN-γ (T/I) for 24 h. Protein expression levels of JNK, p-JNK, p38 MAPK, and p-p38 MAPK in HaCaT cells were determined using Western blotting analysis. GAPDH was used as a reference and the amounts of different proteins were normalized to that of GAPDH. Data are presented as a percentage of control group values (mean ± SD of three to six independent experiments). ** *p* < 0.01 and * *p* < 0.05 indicate a significant difference.

TNF-α/IFN-γ also did not affect the expression levels of total stress-responsive c-Jun N-terminal kinase (JNK) and p38 MAPK but increased their phosphorylation levels by 120% and 789%, respectively. AR extract (0.15 to 1.5 mg/mL) did not affect total and phosphorylated JNK and p38 MAPK protein levels. In addition, AR extract did not affect TNF-α/IFN-γ-induced phosphorylation of JNK and p38 MAPK (Figure 5).

**Figure 6:**
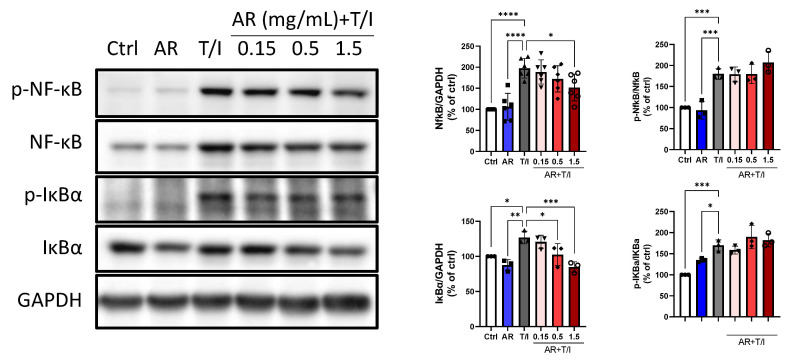
Effects of *Amauroderma rugosum* (AR) extract on total and phosphorylated protein levels of NF-κB and IκBα in HaCaT keratinocytes. HaCaT cells were treated without (control) and with different concentrations of AR extract (0.15 to 1.5 mg/mL) in the presence of 10 ng/mL recombinant human TNF-α and 10 ng/mL IFN-γ (T/I) for 24 h. Protein expression levels of NF-κB, p-NF-κB, IκBα, and p-IκBα in HaCaT cells were determined using Western blotting analysis. GAPDH was used as a reference and the amounts of different proteins were normalized to that of GAPDH. Data are presented as a percentage of control group values (mean ± SD of three to six independent experiments). **** *p* < 0.0001, *** *p* < 0.001, ** *p* < 0.01, and * *p* < 0.05 indicate a significant difference.

TNF-α/IFN-γ increased the expression levels of total nuclear factor-kappa B (NF-κB) and nuclear factor of kappa light polypeptide gene enhancer in B-cells inhibitor alpha (IκBα) by 97% and 27%, respectively. TNF-α/IFN-γ also increased the phosphorylation levels of NF-κB and IκBα by 80% and 70%, respectively. AR extract (1.5 mg/mL) alone did not affect the total and phosphorylated protein levels of NF-κB or IκBα. However, 1.5 mg/mL AR extract reduced the TNF-α/INF-γ-induced total protein levels of NF-κB by 23%, and 0.5 and 1.5 mg/mL AR extract inhibited TNF-α/INF-γ-induced total protein levels of IκBα by 19% and 33%, respectively. Nonetheless, AR extract did not affect TNF-α/INF-γ-induced phosphorylation of NF-κB or IκBα (Figure 6).

**Figure 7:**
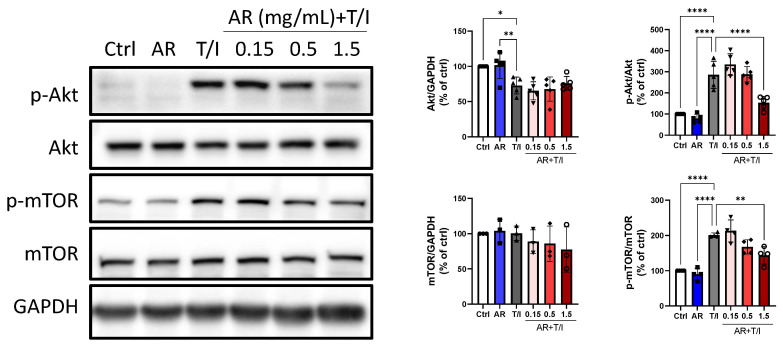
Effects of *Amauroderma rugosum* (AR) extract on total and phosphorylated protein levels of Akt and mTOR in HaCaT keratinocytes. HaCaT cells were treated without (control) and with different concentrations of AR extract (0.15 to 1.5 mg/mL) in the presence of 10 ng/mL recombinant human TNF-α and 10 ng/mL IFN-γ (T/I) for 24 h. Protein expression levels of Akt, p-Akt, mTOR, and p-mTOR in HaCaT cells were determined using Western blotting analysis. GAPDH was used as a reference, and the amounts of different proteins were normalized to that of GAPDH. Data are presented as a percentage of control group values (mean ± SD of three to six independent experiments). **** *p* < 0.0001 ** *p* < 0.01, and * *p* < 0.05 indicate a significant difference.

Lastly, TNF-α/IFN-γ slightly decreased the expression levels of protein kinase B (Akt) by 27%, but it had no effect on the mammalian target of rapamycin (mTOR). TNF-α/IFN-γ increased the phosphorylation levels of Akt and mTOR by 186% and 101%, respectively. AR extract (0.15 to 1.5 mg/mL) alone had no effect on total and phosphorylated protein levels of Akt and mTOR, but AR extract (1.5 mg/mL) reduced the TNF-α/IFN-γ-induced phosphorylation levels of Akt and mTOR by 46% and 28%, respectively (Figure 7).

### 2.6. Drug-Likeness of Gallic Acid, Guanosine, and Uridine

**Figure 8:**
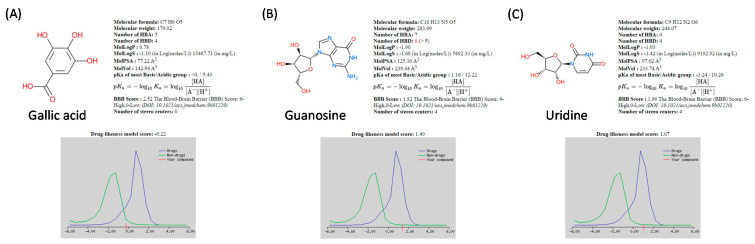
Maximum drug-likeness model score of (**A**) gallic acid, (**B**) guanosine and (**C**) uridine using MolSoft.

Drug-likeness of gallic acid, guanosine, and uridine, which might be the potential active ingredients of AR extract, was evaluated using Molsoft scheme (Figure 8). The drug-likeness value of gallic acid was −0.22, which was not in the drug-like range, whereas the drug-likeness values of guanosine and uridine were 1.40 and 1.07, respectively, which were within the drug-like range.

## 3. Discussion

It has been suggested that AR possesses anti-inflammatory properties. In Asian countries, such as China, AR is used as a medicinal herb for the treatment of acute or chronic nephritis [9]. Moreover, an in vitro study has reported that AR can reduce the gene expression of TNF-α in RAW264.7 cells [4]. Based on this, our study was designed to examine whether the aqueous extract of AR exerts anti-inflammatory effects on keratinocytes.

IL-1β and IL-6 are the most well-known cytokines that mediate inflammatory cell migration, keratinocyte proliferation, and production of additional cytokines by keratinocytes [1]. The most representative inflammatory chemokines, such as macrophage-derived chemokine, RANTES (or regulated upon activation, normal T-cell expressed and secreted), and TARC (or thymus- and activation-regulated chemokine), can regulate the recruitment of leukocytes to the inflammatory site. Other chemokines such as MCP-1 and IL-8 are also closely linked to the initiation and severity of chronic skin inflammation [1]. TNF-α and IFN-γ can synergistically induce the expressions of inflammatory cytokines (IL-6, IL-8, and IL-1β) and chemokines (TARC, MDC, and RANTES) in HaCaT keratinocytes [11]. Therefore, TNF-α/IFN-γ-stimulated HaCaT keratinocytes have been commonly adopted as an in vitro model of inflammatory skin diseases such as atopic dermatitis. The results of the present study showed that the aqueous extract of AR inhibited the production of cytokine IL-1β, and chemokines IL-8 and MCP-1 in TNF-α/IFN-γ-stimulated keratinocytes.

TNF-α/IFN-γ activates several intracellular signaling pathways in keratinocytes, including those related to mitogen-activated protein kinases (MAPK) in HaCaT cells [12]. There are three major groups of MAPK: mitogen-responsive ERK (extracellular signal regulated kinases), stress-responsive JNK/SAPKs (c-Jun N-terminal kinase/stress activated protein kinases), and p38 MAPK. In addition to triggering the release of cytokines and chemokines, these MAPK also control a variety of physiological processes. For instance, the activation of JNK, which can decrease filaggrin expression, is essential for skin hydration and integrity of the skin barrier [13]. ERK regulate proliferation and survival, whereas p38 MAPK affects the differentiation and apoptosis of keratinocytes [14]. Our previous study demonstrated the inhibitory effects of AR on the MEK/ERK-dependent pathways in 6-hydroxydopamine-induced PC12 cells. In this study, the aqueous extract of AR effectively decreased the activation of ERK signaling pathway in TNF-α/IFN-γ-stimulated HaCaT keratinocytes. However, no modulatory effects were observed on JNK or p38 MAPK. Not surprisingly, not all MAPK pathways were inhibited by AR. A similar situation was observed for 3,4,5-trihydroxycinnamic acid, which could reduce skin inflammation through the ERK-dependent pathway [15] but did not exert a regulatory effect on JNK [16] and p38 MAPK [17] activation.

NF-κB, a downstream molecule of MAPK, regulates the expression of genes, enzymes, and adhesion molecules involved in chronic inflammatory diseases [18]. In the cytosol, NF-κB dimers are in an inactive state, bound to IκBα, a natural NF-κB inhibitor. Phosphorylation and release of IκBα leads to the activation of NF-κB, followed by the nuclear translocation of NF-κB p65 [19], which increases the expression of different inflammation-mediated genes [20]. Accumulating evidence indicates that the PI_3_K-Akt-mTOR signaling pathway plays a crucial role in the normal cutaneous healing process, and rates of wound healing are directly dependent on the sustained activation of this pathway [21]. In our study, TNF-α/IFN-γ induced the expression of NF-κB and IκBα, and phosphorylations of Akt and mTOR, whereas AR effectively suppressed these events. Taking all our results into consideration, we believe that aqueous extract of AR may inhibit both MEK/ERK and Akt/mTOR signaling pathways, which in turn inhibit NF-κB-dependent inflammatory response. MEK/ERK and Akt/mTOR are two independent signaling pathways on the upstream of NF-κB-dependent pathway, inhibition of MEK/ERK and Akt/mTOR signaling pathways should result in the decreased activation of NF-κB-dependent pathway. Interestingly, our results showed that AR aqueous extract had a negligible effect on the ratio of phosphorylation of NF-κB/total protein of NF-κB. A possible explanation was that the inhibition of MEK/ERK and Akt/mTOR signaling pathways by AR extract could reduce the phosphorylation of NF-κB and, at the same time, downregulate the transcription factors, which might lead to the decreased protein expression of NF-κB. Both the reduced activation and lowered expression of NF-κB could indeed inhibit the inflammatory response.

Oxidative stress is a key factor contributing to inflammatory disorders. ROS can activate NF-κB, which regulates genes such as TNF-α, interleukins, and inducible nitric oxide synthase (iNOS) [22]. In guinea pig skin, the inhibition of endogenous NO● production can suppress leukocyte accumulation by different inflammatory mediators. These results suggest that NO● plays a crucial role as a neutrophil chemoattractant in skin [23]. Protein kinase C (PKC) is also involved in the interaction between ROS and cytokines. Oxidative stress is known to activate PKC [24], which can increase the expression of TNF-α [25] and the production of cytokines in keratinocytes [26]. Antioxidant compounds have been widely proposed for the prevention of various skin disorders [27,28]. Antioxidant effect is the most reported bioactivity of AR [9]. Consistently, the present study also demonstrated the antioxidant effects of AR aqueous extract in HaCaT keratinocytes. The antioxidant effect of the aqueous extract of AR is likely due to its capacity to quench DPPH• free radicals and Cu^2+^ radicals [5,7]. Apart from its direct scavenging activities on ROS, other mechanisms such as the activation of the nuclear factor (erythroid 2)-related factor 2/heme-oxygenase-1 signaling pathway may also be involved. Our recent study demonstrated that AR could activate this pathway, which may protect cardiomyocytes from DOX-induced oxidative stress [29].

The total content of polysaccharide, triterpene, and phenolic compound in the aqueous extract of AR were measured in our study. Our previous study also reported that the content of total phenolic compounds in the aqueous AR extract was higher than that in the aqueous extract of *Ganoderma lucidum* [29]. This may explain why AR is stronger than *Ganoderma lucidum* in protecting cardiomyocytes from DOX-induced oxidative stress [29]. According to our mass spectrometry results, gallic acid was the major phenolic compound in the aqueous extract of AR. Gallic acid can eliminate free radicals and inflammatory molecules [30,31,32]. Moreover, its anti-inflammatory properties have been demonstrated in various tissues. For instance, gallic acid suppresses the levels of cytokines IL-1 and IL-6, chemokines MCP-1 and MCP-3, cyclooxygenase-2 (COX-2), and matrix metalloproteinase-9 (MMP-9) in fibroblast-like synoviocytes in rheumatoid arthritis [33]. In a mouse model of allergic rhinitis, gallic acid decreased the IL-4, IL-13, and IL-17 levels in mouse model of allergic rhinitis [31]. Interestingly, oral administration of gallic acid reduced the levels of TNF-α, IL-4, IFN-γ, and IL-17 in the lymph nodes of a 4-dinitrochlorobenzene-induced mouse model of skin inflammation [34]. In the same model, gallic acid also activated regulatory T cells to suppress the immune response via the production of IL-10 and TGF-β [34]. The effects of gallic acid on HaCaT keratinocytes were evaluated in this study. The concentration of AR extract that exerted anti-inflammatory effect was 1.5 mg/mL, which contained 10 µM of gallic acid. However, we found that this concentration of gallic acid could only reduce IL-1β release but could not significantly suppress the release of IL-8 and MCP-1 in HaCaT keratinocytes. Therefore, gallic acid alone could not account for the anti-inflammatory effects of AR.

In addition to gallic acid, the aqueous extract of AR also contained nucleosides such as guanosine and uridine, which are known to possess anti-inflammatory properties. For instance, uridine reduced oedema and infiltration of leukocytes in the bronchoalveolar lavage fluid in an animal model of lung inflammation [35,36]. Moreover, administration of guanosine or uridine reduced cytokines such as IL-4, IL-6, IL-13, and ovalbumin-specific IgE in the lungs of OVA-challenged asthmatic mice. In addition, the production of IL-6 and the expression of key molecules in the MAPK and NF-κB pathways were decreased by uridine and guanosine in lipopolysaccharide/IFN-γ-induced THP-1 cells and lung tissues [37]. In a dinitrobenzene sulfonic acid-induced colitis model, guanosine lowered IL-1β, IL-6, and TNF-α mRNA levels and downregulated the expression of NF-κB p65 and the levels of ROS and nitrite [38]. Treatment with uridine prevented lipopolysaccharide-induced increases in TNF-α, TNF-γ, IL-1, IL-2, and IL-6 and suppressed NF-κB signaling pathway activity in mouse spleen lymphocytes [39]. In the brain, guanosine inhibits lipopolysaccharide-induced augmentation of pro-inflammatory cytokine levels, NF-κB activation, mitochondrial dysfunction, and oxidative stress in hippocampal astrocytes. These effects are mediated through activation of the heme oxygenase-1 pathway [40]. Moreover, guanosine treatment reduced infarct volume and attenuated the increase in inflammatory parameters such as IL-1, IL-6, IL-10, TNF-α, and INF-γ in the brain tissue and cerebrospinal fluid under ischemic conditions [41]. Finally, local administration of uridine in the joint could prevent the development of antigen-induced arthritis, probably through downregulation of adhesion molecule expression, decreased production of TNF-α and IL-6, and inhibition of leukocyte extravasation [42].

According to the results of our chemical analysis, 5 µM guanosine and uridine were found in 1.5 mg/mL of the AR extract. However, this concentration of guanosine or uridine only reduced IL-1β and IL-8 release but did not affect the release of MCP-1 in HaCaT keratinocytes. Therefore, similar to gallic acid, guanosine or uridine alone did not contribute to the anti-inflammatory effect of AR extract on HaCaT cells. Interestingly, the combination of gallic acid, guanosine, and uridine could inhibit the release of IL-1β, IL-8 and MCP-1 in HaCaT cells. Nevertheless, the effect of combination of gallic acid, guanosine and uridine on IL-8 release was higher than that of AR extract. It clearly indicated that other active ingredients may be present and able to inhibit IL-8 release. In contrast, the effects of combination of gallic acid, guanosine and uridine on IL-1β and MCP-1 release were lower than that of AR extract. The reason is not known. Further investigation is required to verify if there are some compounds in AR extract that may trigger IL-1β and MCP-1 release.

As mentioned above, we cannot exclude the possibility that other compounds present in the aqueous extract of AR (Table 1) may also contribute to its anti-inflammatory effect. For instance, it has been reported that oleamide can reduce the expression of inflammatory mediators such as iNOS, COX-2, and pro-inflammatory cytokines in lipopolysaccharide-stimulated RAW264.7 macrophages [43]. Moreover, ganoderic acid A and ganoderic acid J, which are found in the aqueous extract of AR, are also known to reduce TNF-α production by RAW 264.7 macrophages [44]. The effects of these compounds have not been studied here because they are not so water soluble and their content in water extract of AR are low. It is hoped that more potential anti-inflammatory components can be identified in AR through further studies.

Topical corticosteroids are still the mainstay of treatment for inflammatory skin diseases, such as atopic dermatitis [45]. However, their adverse effects, such as cutaneous atrophy, skin thinning, and immunosuppression, make them unsuitable for long-term use [46]. Tacrolimus, a calcineurin inhibitor with immunosuppressive effects, is an alternative to topical corticosteroids [47]. It is particularly useful in thin-skin areas such as the face and flexures [47]. However, tacrolimus causes local reactions such as skin burning sensation and systemic adverse effects such as hypertension, nephrotoxicity, and renal injury [48,49]. Therefore, it is necessary to identify an alternative cure for inflammatory skin diseases with a better safety profile. Extracts of herbal plants such as AR, as shown in this study, may be promising agents. The present study was conducted in a cell model. However, it of interest to note that guanosine and uridine, which are the possible active ingredients in AR, possess favorable pharmacokinetic properties as reflected from the drug-likeness score in Molsoft schcme. Therefore, animal studies and clinical trials for further verifying the usefulness of AR in treating inflammatory skin diseases are highly encouraged.

## 4. Materials and Methods

### 4.1. Chemicals and Reagents

RPMI 1640 medium, penicillin-streptomycin, 0.25% trypsin, fetal bovine serum, human TNF-α recombinant protein, and CM-H2DCFDA were purchased from Invitrogen (Carlsbad, CA, USA). RIPA Lysis and Extraction Buffer, Halt™ Protease Inhibitor Cocktail, and BCA Protein Assay Kit were purchased from Thermo Fisher Scientific (Waltham, MA, USA). Gallic acid, guanosine, uridine, thiazolyl blue tetrazolium bromide (MTT), dimethyl sulfoxide (DMSO), and phenylmethylsulfonyl fluoride (PMSF) were purchased from Sigma-Aldrich (St. Louis, MO, USA). The human CXCL8/IL-8 DuoSet ELISA, human CCL2/MCP-1 DuoSet ELISA, human IL-1β/IL-1 F2 Duoset ELISA, and human IFN-γ recombinant protein were purchased from R & D Systems (Minneapolis, MN, USA). All the antibodies used for Western blotting were purchased from Cell Signaling Technology (Danvers, MA, USA). ECL Select Western blotting detection reagents were purchased from GE Healthcare Life Sciences (Piscataway, NJ, USA).

### 4.2. Reflux Extraction of AR

AR was provided by Hong Kong Ganoderma Centre Limited (Hong Kong, China). The fruiting bodies of the AR were dried and powdered. Two grams of AR powder were boiled in 50 mL distilled water for an hour. The extract was then centrifuged at 4000× *g* for 10 min. The supernatant was then collected. The undissolved residue was boiled in water twice, and all the extracted residues were pooled, filtered, and condensed to 80 mL using a rotavap. The extract was stored at −20 °C. The extract was filtered through membrane filters with pore size of 0.22 µm before experiments.

### 4.3. Determination of Total Polysaccharide, Triterpene, and Phenolic Compounds in AR Extract

The total polysaccharide, triterpene, and phenolic compounds were analyzed by chemical assays, as reported by us previously (Li et al., 2022 [29]). Glucose was used as the standard for polysaccharide measurements. The results are expressed as milligram glucose equivalent per gram (mg GE/g). Oleanolic acid was used as the standard for the triterpene measurements. The results are expressed as milligram oleanolic acid equivalent per gram (mg OAE/g). Gallic acid was used as a standard for the measurement of phenolic compounds. All determinations are expressed as milligram gallic acid equivalents per gram (mg GAE/g).

### 4.4. Qualitative and Quantitative Measurement of Compounds in AR Extract

The compounds in the AR extract were identified using ultra-performance liquid chromatography (UPLC) Q-Exactive Orbitrap tandem mass spectrometry (MS) (Thermo Fisher Scientific Inc., Waltham, MA, USA) fitted with an electrospray ionization source. The following experimental parameters were adopted: ion spray voltage, 3.5 kV; capillary temperature, 320 °C; sheath gas flow rate, 35 Arb; heater temperature, 350 °C; auxiliary gas flow rate, 10 Arb. Data were collected for both positive and negative ions with a mass scanning range of 50–1500 m/z. Data were processed and analyzed using Xcalibur™ version 2.2.1 and Trace Finder 3.3 version (Thermo Fisher Scientific Inc.) along with compound discoverer software (version 3.0; Thermo Fisher Scientific Inc.).

A Dionex UltiMate 3000 Rapid Separation UPLC system (Thermo Fisher Scientific Inc.) equipped with a DAD-3000RS detector was used for the quantitative analysis. The chromatographic separation was carried out at 30 °C on a SunFire C_18_ column (Waters, MA, USA). The mobile phase A contained 0.1% formic acid (*v*/*v*) in water and mobile phase B contained and 0.1% formic acid (*v*/*v*) in acetonitrile. The flow rate was 0.30 mL/min, and the injection volume was 2 µL. The gradient elution processes were 0–45 min, 5–95% B; 45–55 min, 95% B. UV-visible spectra were recorded in the range of 190–400 nm. Different reference standards were used according to the results of UPLC Q-Exactive Orbitrap tandem MS.

### 4.5. Cell Culture and Drug Treatment

HaCaT keratinocytes were purchased from AddexBio (San Diego, CA, USA). The cells were cultured in RPMI medium supplemented with 8% heat-inactivated FBS, 1% penicillin-streptomycin, and cultured at 37 °C in an incubator with a humidified atmosphere of 5% CO_2_.

HaCaT cells were treated with AR extract (0–1.5 mg/mL) in the presence of 10 ng/mL recombinant human TNF-α and 10 ng/mL IFN-γ for 24 h. Inflammatory responses, reactive oxygen species levels, and signaling pathways were studied.

### 4.6. Cell Viability Assay

To study the toxicity of AR extract in HaCaT cells, cell viability was assessed using the MTT assay. After the drug treatment, the cells were incubated with 0.4 mg/mL MTT for 3 h at 37 °C. DMSO was then added to lyse the cells and dissolve the purple formazan crystals formed inside the cells. Absorbance was measured using a microplate reader at 560 nm.

### 4.7. Enzyme-Linked Immunosorbent Assay (ELISA)

After the treatment with AR extract, the culture media were collected and centrifuged at 5000× *g* at 4 °C for 10 min to remove the cell debris. Interleukin (IL)-1β, IL-8, and monocyte chemoattractant protein (MCP)-1 in the supernatants were detected using the human IL-1β/IL-1 F2 Duoset ELISA, human CXCL8/IL-8 DuoSet ELISA, and human CCL2/MCP-1 DuoSet ELISA, respectively, according to the manufacturer’s instructions.

### 4.8. ROS Measurement

The cells were then washed with PBS and stained with 2 µM CM-H2DCFDA for 15 min. After thorough washing with PBS to remove the unbound dyes, the cells were detached from the culture plate using 0.25% trypsin. The green fluorescent signal of CM-H2DCFDA was examined via flow cytometry using the FITC channel.

### 4.9. Western Blot Analysis

Protein of HaCaT keratinocytes were extracted using RIPA lysis buffer containing 1% PMSF and 1% Halt™ Protease Inhibitor cocktail. The cell lysate was centrifuged at 13,000× *g* at 4 °C for 10 min. The supernatant was then collected. To determine the total protein concentration of each sample, BCA assay was performed according to the manufacturer’s instructions. Equal amounts of protein samples were loaded onto 8–10% SDS gel for electrophoresis and then electrotransferred to PVDF membranes. The membranes were blocked with 5% non-fat milk for 1 h at room temperature and then incubated with primary antibodies against ERK1/2, phospho-ERK1/2 (Thr202/Tyr204), MEK1/2, phospho-MEK1/2 (Ser217/221), JNK, phospho-JNK (Tyr183/Tyr185), p38, phospho-p38 (Thr180/Tyr182), NF-κB p65, phospho-NF-κB p65 (Ser536), Akt, phospho-Akt (Ser473), mTOR, phospho-mTOR (Ser2448), or GAPDH (1:1000 dilution in 5% BSA in TBS) at 4 °C overnight. After washing thrice with TBS containing 0.1% Tween-20 (TBST), the membrane was subjected to horseradish peroxidase-conjugated secondary antibody (1:2500 dilution in 5% BSA in TBS) for 1 h at room temperature. The membrane was washed again with TBST and visualized using ECL Select Western blotting Detection Reagents with a ChemiDoc XRS Molecular Imager (Bio-Rad Laboratories, Hercules, CA, USA).

### 4.10. Quantification of Drug-Likeness

Molsoft (http://molsoft.com/mprop/ accessed on 25 September 2022) was used for quantifying the drug-likeness of compounds. Based on various molecular properties, including the number of hydrogen bond acceptor (HBA), number of hydrogen bond donor (HBD), molecular weight, water partition coefficient (MolLogP), water solubility (MolLogS), molecular polar surface area (MolPSA), molecular polar surface volume (MolVol), acid dissociation constant (pKa), and blood–brain barrier (BBB) score, the software quantified a value known as “Molsoft Score”, which determines the drug-likeness of compounds.

### 4.11. Statistical Analysis

All data are expressed as the mean ± SD of at least three independent experiments. One-way ANOVA followed by Dunnett’s multiple comparisons test in the GraphPad Prism 9 software (GraphPad Software Inc., San Diego, CA, USA) was used to calculate statistical significance within groups. Statistical significance was set at *p* < 0.05.

## 5. Conclusions

In summary, our study demonstrated that the aqueous extract of AR exerts anti-inflammatory effects on HaCaT keratinocytes, probably through its antioxidant effect and the inhibition of MEK/ERK, Akt/mTOR, and NF-κβ signaling pathways. Our results also showed that the aqueous extract of AR contains gallic acid, guanosine, and uridine. They may be part of the active ingredients responsible for the anti-inflammatory effects of AR, although the presence of other active ingredients cannot be excluded. Further studies using animal models and clinical trials are required to confirm the potential application of AR in treating inflammatory skin diseases.

## Figures and Tables

**Table 1:** Characterization of compounds from AR extract by UPLC-Q-Exactive Orbitrap/MS analysis.

Name	Formula	Molecular Weight	Retention Time (min)	Observed (m/z)	Theoretical (m/z)	Ion Type	Mass Error (ppm)	Fragment Ions
Ganoderic acid A	C30H44O7	516.3095	23.52	515.3010	515.3014	[M-H]−	−0.78	515.301, 497.2911, 435.2890, 299.1651, 285.1497, 195.1020
Ganoderic acid D	C30H42O7	514.2927	22.93	515.3011	515.3003	[M+H]+	1.55	515.2990, 497.2875, 479.2782, 461.2675, 451.2803
Ganoderic acid J	C30H42O7	514.2927	24.36	515.3008	515.3003	[M+H]+	0.97	515.3000, 497.2881, 479.2786, 461.2677
Oleamide	C18H35NO	281.2718	46.60	282.2785	282.2791	[M+H]+	−2.13	282.2785, 265.2520
(9Z, 12Z)-octadeca-9, 12-dienamide	C18H33NO	279.2559	43.02	280.2626	280.2635	[M+H]+	−3.21	280.2626, 263.2363, 245.2259
Gallic acid	C7H6O5	170.0211	5.44	169.0136	169.0142	[M-H]−	−3.55	169.0136, 125.0235
Adenosine	C10H13N5O4	267.0972	3.31	268.1035	268.1040	[M+H]+	−1.86	268.1035, 136.0619
Uridine	C9H12N2O6	244.0698	3.52	243.0624	243.0623	[M-H]−	0.41	-
Guanosine	C10H13N5O5	283.0919	4.10	282.0846	282.0844	[M-H]−	0.71	282.0846, 150.0415
Adenine	C5H5N5	135.0544	1.93	136.0619	136.0618	[M+H]+	0.73	136.0619, 119.0355
Cytosine	C4H5N3O	111.0437	1.73	112.0509	112.0505	[M+H]+	3.57	112.0509, 95.0245
Uracil	C4H4N2O2	112.0278	3.16	113.0349	113.0346	[M+H]+	2.65	113.0349, 96.0086, 70.0295
Dextrose	C6H12O6	180.0631	2.45	179.0557	179.0561	[M-H]−	−2.23	179.0557, 119.0341
L-Arabinopyranose	C5H10O5	150.0523	2.92	149.0449	149.0455	[M-H]−	−4.03	-

## Data Availability

The data that support the findings of this study are available from the corresponding author, George Pak-Heng Leung, upon reasonable request.

## Sample Availability

Samples of the *Amauroderma rugosum* are available from the corresponding author, George Pak-Heng Leung, upon reasonable request.
